# Rapid Method of Wastewater Classification by Electronic Nose for Performance Evaluation of Bioreactors with Activated Sludge †

**DOI:** 10.3390/s23208578

**Published:** 2023-10-19

**Authors:** Magdalena Piłat-Rożek, Marcin Dziadosz, Dariusz Majerek, Katarzyna Jaromin-Gleń, Bartosz Szeląg, Łukasz Guz, Adam Piotrowicz, Grzegorz Łagód

**Affiliations:** 1 Faculty of Mathematics and Information Technology, Lublin University of Technology, 20-618 Lublin, Poland; m.pilat-rozek@pollub.pl (M.P.-R.); m.dziadosz@pollub.pl (M.D.); d.majerek@pollub.pl (D.M.); 2 Institute of Agrophysics, Polish Academy of Sciences, 20-290 Lublin, Poland; k.jaromin-glen@ipan.lublin.pl; 3 Institute of Environmental Engineering, Warsaw University of Life Sciences—SGGW, 02-797 Warsaw, Poland; bartosz_szelag@sggw.edu.pl; 4 Faculty of Environmental Engineering, Lublin University of Technology, 20-618 Lublin, Poland; l.guz@pollub.pl (Ł.G.); a.piotrowicz@pollub.pl (A.P.)

**Keywords:** electronic nose, multidimensional data analysis, principal component analysis, DBSCAN algorithm, extra trees, wastewater classification, performance evaluation, activated sludge

## Abstract

Currently, e-noses are used for measuring odorous compounds at wastewater treatment plants. These devices mimic the mammalian olfactory sense, comprising an array of multiple non-specific gas sensors. An array of sensors creates a unique set of signals called a “gas fingerprint”, which enables it to differentiate between the analyzed samples of gas mixtures. However, appropriate advanced analyses of multidimensional data need to be conducted for this purpose. The failures of the wastewater treatment process are directly connected to the odor nuisance of bioreactors and are reflected in the level of pollution indicators. Thus, it can be assumed that using the appropriately selected methods of data analysis from a gas sensors array, it will be possible to distinguish and classify the operating states of bioreactors (i.e., phases of normal operation), as well as the occurrence of malfunction. This work focuses on developing a complete protocol for analyzing and interpreting multidimensional data from a gas sensor array measuring the properties of the air headspace in a bioreactor. These methods include dimensionality reduction and visualization in two-dimensional space using the principal component analysis (PCA) method, application of data clustering using an unsupervised method by Density-Based Spatial Clustering of Applications with Noise (DBSCAN) algorithm, and at the last stage, application of extra trees as a supervised machine learning method to achieve the best possible accuracy and precision in data classification.

## 1. Introduction

The operational properties of wastewater treatment plant (WWTP) facilities should be adjusted to the influent parameters, so that wastewater quality indicators can be maintained at an appropriate level and the stringent regulations can be met. This requires conducting regular measurements [1,2,3,4]. Currently, there are multiple devices and methods for assessing wastewater parameters in the form of basic indicators, such as biochemical oxygen demand (BOD), chemical oxygen demand (COD), total organic carbon (TOC), oxygen uptake rate (OUR), total suspended solids (TSS), and volatile suspended solids (VSS), in addition to the levels of phosphorus and nitrogen compounds [3,5]. Despite a marked improvement in the automation of treatment processes in recent years, a system enabling automatic, online measurement of important wastewater parameters is still lacking. Determination of the aforementioned parameters using appropriate techniques may take from 1 h (COD) up to 5 d (BOD_5_) [6,7,8]. Moreover, professional measuring equipment is prohibitively expensive. Therefore, measurements in many small-sized WWTPs are seldom conducted, whereas treatment is carried out based on staff observations, relying on their senses and practical knowledge.

The values of measured pollution indicators are directly related to the properly conducted and highly efficient process of removing pollutants in activated sludge bioreactors. In virtually every treatment system, emerging process malfunctions become apparent with high levels of pollutant indicators and their exceeded limits under current legal norms.

Alternatively, in many WWTPs, online estimation of wastewater parameters can be performed using electronic noses. Currently, e-noses are used for measuring odorous compounds; these devices mimic the mammalian olfactory sense, comprising an array of multiple non-specific gas sensors and appropriate data analysis techniques [9]. The most common sensors used in e-noses include: conducting polymers (CP), surface acoustic wave (SAW) sensors, quartz crystal microbalance (QCM), and metal oxide semiconductor (MOS) resistance sensors [10]. Particular sensors are partially sensitive to different types of chemical compounds [4]. Therefore, the sensors employed in e-noses should be sensitive to different groups of pollutants.

The gas sensor arrays provide a repeatable signal, and the e-nose manufactured on their basis does not adapt to noxious odors, unlike the human olfactory sense, since the array can be quickly flushed with clean air as a standard.

In contrast to chromatographic techniques, the gas sensors in e-noses are unable to accurately (quantitatively and qualitatively) identify individual chemical compounds. In order to obtain satisfactory results, it is necessary to employ multiple sensors, since a particular signal can be generated by several different gas samples.

Each gas mixture generates a distinctive signal profile that may be likened to fingerprints in dactyloscopy, since it is highly improbable that two different gas samples will yield the same combination. Thus, the term “gas fingerprint” is frequently employed when considering different signal combinations. Appropriate statistical analyses of multidimensional data are conducted for this purpose, such as artificial neural networks (ANN) [11,12], decision trees (DT) and random forests (RF) [13], support vector machines (SVM) [14], t-distributed stochastic neighbor embedding (t-SNE) [15], cluster analysis (CA) methods, or principal component analysis (PCA) [16].

Attempts have been made to conduct a comparison of standard wastewater parameters to e-nose response [17,18]. These have involved assessing the e-nose system in terms of recognition and classification of wastewater odors regarding their location in a wastewater treatment plant, as well as evaluation of odor concentration. Previous studies attempted to utilize the e-nose to assess standard physical-chemical parameters of wastewater (e.g., such as volatile organic compounds (VOC) [17], COD, BOD [19,20], turbidity, VSS, and TSS [17]).

Unfortunately, due to the complexity of the relationships between gas sensor array readings, using deterministic models for the classification of objects is not sufficient. However, high classification capability can be achieved using an appropriate advanced machine learning model [15].

Taking into account the concentration and content of VOCs in wastewater, the obtained profiles might be highly diversified. Up to 450 compounds can be found in the gases emitted during the treatment of wastewater; approximately 100 of them are strong odorants [21], characterized by a wide range of odors. The concentration of VOCs above the water surface is closely related to the concentration of pollutants in wastewater. The pollution levels can be reduced via three different methods (i.e., biodegradation, sorption on solid surfaces, and volatilization) [22]. Volatilization is primarily related to volatile organic compounds [23], polycyclic aromatic hydrocarbons [24], surfactants [25], and other pollutants (e.g., phenol, hydrogen sulfide, and acetone) [26]. Process intensity is highly dependent on the operating conditions, namely aeration and mixing, pressure, temperature, as well as disturbances of standard technological processes. As a result of air, raising the mass transfer of pollutants between phases, volatilization by stripping occurs in the aeration tank.

The failures of the wastewater treatment process are directly related to the odor nuisance of the bioreactors and are reflected in the level of pollution indicators. An e-nose may be used to identify the gas pollutants that are emitted during wastewater treatment. Most frequently, this process includes evaluating the possibility of using e-noses for the classification and identification of odors depending on the place of their origin (in WWTP, sewer system, or surface water) [27,28], as well as assessing the odor concentration in the studied samples of air [29,30,31]. The above-mentioned papers made an assumption that heavily polluted wastewater should be distinct from wastewater polluted to a lesser degree. Thus, e-noses may be employed for the early detection of detrimental chemical compounds, which could possibly disrupt the activity of microorganisms in the biological part of a wastewater treatment plant. For example, crude oil derivatives, which are hardly biodegradable, negatively impact activated sludge performance, disrupting the treatment process [18].

Failures in the activated sludge treatment process can also result from malfunctions of the systems ensuring proper conditions in the zones of the bioreactor chambers in the case of flow-through systems, or during specific phases of sequencing batch reactor (SBR) operation. As the name implies, the concept of SBR technology is to treat wastewater using the activated sludge, where the processes of biological treatment and separation occur in the same tank in a sequential mode. SBRs are most popular as solutions in urban areas, as well as in rural areas [32,33,34,35]. Examples of malfunctions include failures of aeration systems resulting in the disruption of aerobic conditions, or failures of mixing systems necessary for proper processes under hypoxic and anaerobic conditions. A gas sensor array may be employed for classifying an abnormal situation, since it enables one to identify numerous types of pollutants.

A few publications show that e-noses are well suited to indicate the problematic situations related to the operation of activated sludge bioreactors [4,36,37]. Thus, it can be assumed that appropriately selected methods of multivariate data analysis will be able to distinguish and classify the operating states of bioreactors. This includes differentiating between phases of normal operation (e.g., the phase involving the introduction of a batch of raw wastewater, treated wastewater ready to be discharged from the bioreactor after the treatment process), as well as indicating the occurrence of insufficiently aerobic conditions associated with, for example, a failure of the aeration process. The team’s previous work has focused primarily on visualizing the data related to stable bioreactor operation and emerging failure conditions, as well as restoring normal operating conditions of the activated sludge [36]. The current work focuses on developing a proposal for a complete protocol for analyzing the visualization and interpretation of multidimensional data from a gas sensor array measuring the properties of the headspace air in a bioreactor. These include dimensionality reduction and visualization in two-dimensional space, application of data clustering using an unsupervised method, and at the last stage, usage of supervised machine learning methods to achieve the best possible accuracy and precision in data classification. The methods presented were chosen to best deal with the specific properties of the data associated with changing conditions. Classically, data are gathered in one cluster distributed around a certain centroid (calculated, for example, using the mean or median) in a spherical way, but sometimes the data form a line or a chain. Then, for example, clustering methods such as k-means or k-median may fail to detect that the data belong to a single group. However, there are methods, such as Density-Based Spatial Clustering of Applications with Noise (DBSCAN), which, in addition to detecting the typical situations where data are located in close proximity around a certain point, also detect when data are located in chains or different random shapes [38].

An important issue is also the detection of abnormal situations, which are linked both to an emergency and the appearance of an unknown disturbance or chemical potentially harmful to activated sludge microorganisms.

## 2. Machine Learning Methods for Multidimensional Data Analysis

Multidimensional data from gas sensor arrays were visualized using the PCA method, the DBSCAN algorithm was used to classify objects using unsupervised learning, and the extremely randomized trees (extra trees) classifier was used for supervised classification.

Principal component analysis is a method of extracting information from a data set by means of reducing its dimensionality and representing it with new variables that are linear combinations of variables from the original set (i.e., the principal components with the largest possible variance) [39]. This method was independently presented in the works of Pearson [40] and Hotteling [41]. The transformation of variables by the PCA method assigns to a data set X with the averages of its column vectors μ a matrix Y of the form

(1) $$Y=\Gamma^{T}(X-\mu ),$$

where Γ is an orthogonal matrix of principal component factor loadings—the loadings of the i-th principal component are in the i-th column of this matrix. With the Γ and Σ matrices, a diagonal matrix is created:

(2) $$\Lambda =\Gamma^{T}\Sigma \Gamma ,$$

in which the main diagonal contains its eigenvalues

(3) $$\lambda_{1}\geq \lambda_{2}\geq_{\;}\ldots_{\;}\geq \lambda_{n},$$

where n is the number of variables of the set X. The eigenvalues are non-negative as long as the matrix Σ is positive definite [42].

Various criteria are used to select the number of components used for further analysis and visualization, including the Kaiser criterion and the explained variance criterion. In the Kaiser criterion, only principal components with eigenvalues greater than or equal to 1 are considered [43]. In the case of this criterion, a scree plot is also often used to visualize the eigenvalues of individual components. The criterion of explained variance is to select as many first components as possible exceeding the designated threshold of the cumulative percentage of explained variance. This threshold depends on the domain in which the analysis is performed, but also on the data set under consideration. The cut-off may be set from 60% to 90%, depending on the number of variables in the dataset and the dominance of principal components in individual explained variance [44].

As PCA reduces the dimensionality of the data set, it is often used to represent multidimensional data in a graph. The technique has been used in this way to visualize the data from gas sensor arrays [36,45], but also other applications related to environmental engineering [46,47]. The authors of the paper [47] used the PCA method on three datasets of relatively less polluted, medium polluted, and highly polluted sites. Their results were compared to determine the components with an eigenvalue greater than 1. Then, factor analysis with the same number of factors was performed on the determined components. Then, comparisons were made on which variables had the largest absolute value loadings to identify the variables responsible for variations in river water quality.

The non-hierarchical object clustering method DBSCAN is presented in the work [48]. It is a density-based algorithm, the results of which depend on the given input parameters minPts (the minimum number of elements required to form a cluster), $minPts\in Z_{+}$, and ε (the maximum radius of the neighborhood, where ε≥0). In this clustering algorithm, a random point p is selected from the set  S, for which ε-neighborhood is calculated as

(4) $$N_{\varepsilon }\left(p\right)=\left\{q\in S:\;d\left(p,q\right)<\varepsilon \right\},$$

where d is the chosen metric. If the power of the set of ε-neighborhood of a point p is no less than minPts, then such a point is called a core and starts a new cluster, to which all points from its surroundings are attached. The set of all points belonging to the neighborhood of the core is also searched; if any of them is also a core, then the points from its neighborhood are attached to the current cluster. The construction of the current cluster ends when all the points belonging to it have been searched. Next, further points not yet classified are drawn, and their ε-neighborhood is checked. All points that have not been classified into any of the clusters at the end of the algorithm are called noise [48,49].

In order to assess the quality of clustering, the following measures can be analyzed:

- Homogeneity (h), which shows whether created clusters only contain points from one class and completeness (c), which gives the information whether the class observations are assigned to the same cluster. These measures are calculated for sets of classes $C=\left\{c_{i}:\;i=1,2,\ldots ,l\right\}$ and set of clusters resulting from the carried out algorithm $K=\left\{k_{i}:i=1,2,\ldots ,m\right\}$ with the following formulas:
  (5) $$h=\left\{\begin{array}{cc} 1\; & if\;H\left(C,K\right)=0\\ 1-\frac{H(C|K)}{H(C)} & else \end{array}\right.,$$
  (6) $$c=\left\{\begin{array}{cc} 1\; & if\;H\left(K,C\right)=0\\ 1-\frac{H(K|C)}{H(K)} & else \end{array}\right..$$
  The conditional entropies are defined as $H\left(C|K\right)=-\sum_{k=1}^{m}\sum_{c=1}^{l}\frac{n_{c,k}}{N}\cdot \log\frac{n_{c,k}}{n_{k}}$, $H\left(K|C\right)=-\sum_{c=1}^{l}\sum_{k=1}^{m}\frac{n_{c,k}}{N}\cdot \log\frac{n_{c,k}}{n_{c}}$, individual entropies as $H\left(C\right)=-\sum_{c=1}^{l}\frac{n_{c}}{N}\cdot \log\frac{n_{c}}{N}$, $H\left(K\right)=-\sum_{k=1}^{m}\frac{n_{k}}{N}\cdot \log\frac{n_{k}}{N}$, and the joint entropy as $H\left(C,K\right)=H\left(K,C\right)=H\left(C|K\right)+H\left(K\right)=H\left(K|C\right)+H(C)$. Additionally, $n_{c,k}$ is the number of data points from class c assigned to cluster k, $n_{k}$ is the number of observations assigned to class k, $n_{c}$ is the number of observations from class c, and N is the cardinality of the whole dataset [50]. Both measures belong to the set [0,1], where values closer to 1 indicate better clustering performance.
- V-measure is derived from homogeneity and completeness as presented in paper [51] and is calculated as
  (7) $$V_{\beta }=\frac{\left(1+\beta \right)\cdot h\cdot c}{\left(\beta \cdot h\right)+c},$$
  where β is a parameter set by user, which in Python package sklearn version 1.0.2 by default is equal to 1. In addition, $V_{\beta }\in [-1,\;1]$, and the closer the value is to 1, the better the clustering [50].
- Adjusted mutual information is also a measure connected to the entropy measure. The mutual information necessary to calculate this measure is defined as
  (8) $$MI\left(C,K\right)=H\left(C\right)-H\left(C|K\right)=H\left(K\right)-H(K|C),$$
  where H(C) and H(K) are the individual entropies, and H(C|K) and H(K|C) are the conditional entropies defined beforehand for homogeneity and completeness measures. Then, the adjusted mutual information is calculated as
  (9) $$AMI\left(C,K\right)=\frac{MI\left(C,K\right)-E\left\{MI(C,K)\right\}}{\frac{1}{2}\left(H\left(C\right)+H(K)\right)-E\left\{MI(C,K)\right\}},$$
  where $E\left\{MI(C,K)\right\}$ is the expected value of mutual information of classes C and clusters K. The score for the AMI measure reaches a maximum value of 1, where 1 indicates a perfect match [52].
- The adjusted Rand index, as presented by Hubert and Arabie in [53], is also a measure of agreement between the true classes of object (C) and the groups assigned by the clustering method (K). The Rand index is defined as follows:
  (10) $$\mathrm{RI}=\frac{a+b}{C_{2}^{n_{samples}}},$$
  where a is the number of pairs of data points in the same group in C and in the same group in K, b is the number of pairs of data points that are in different groups in C and in different groups in K, and $C_{2}^{n_{samples}}$ is the total number of pairs in the whole data set. The adjusted Rand index is given by the formula:
  (11) $$\mathrm{ARI}=\frac{RI+E[RI]}{\underset{\;}{\max}\left(RI\right)-E[RI]},$$
  where E[RI] is the expected value of RI. The score for the ARI measure is between −0.5 and 1, where 1 indicates a perfect match [54].
- The last measure is the silhouette coefficient, as presented in [55], which can be counted for an i-th observation in the dataset as
  (12) $$s(i)=\frac{b\left(i\right)-a(i)}{\underset{\;}{\max}\left\{a\left(i\right),\;b(i)\right\}},$$
  where a(i) is the average distance between the given point and all the other points in the sample cluster, and b(i) is the average distance between the point and all the other points in the next closest on the average cluster. To obtain the silhouette coefficient for the entire dataset, the arithmetic mean of all s(i) values is calculated. Its values are from the set [−1,1], where −1 indicate the worst possible clustering, near 0 mean that the clusters are overlapping, and 1 points to the fact that the obtained clustering is the best [50].

The DBSCAN algorithm has been used for the purposes of grouping areas of a municipal water supply network into water leakage risk groups [56], detecting outlier observations from IoT sensors for the identification of automotive failures [57], and classifying data from wastewater monitoring systems for anomaly detection [58].

Extra trees is a decision-tree-based method that can be applied to both regression and classification tasks. The algorithm was first described in 2006 by Geurts et al. in the paper [59]. In this method, each of the M trees is trained on the entire learning set. In the case of classification trees, splitting rules at each node of the tree are created until the power of the set of elements in that node falls below the minimum number $n_{min}$ or the node contains observations from multiple classes of the outcome variable. These rules in extra trees are created so that K of all the explanatory variables in each node are drawn. Using each of these variables $a_{i}$, where $i\in \left\{1,2,\ldots ,K\right\}$, a split is created at that node. If $a_{i}$ is a numerical variable, then its minimum $a_{min\;}^{S}$ and maximum $a_{max}^{S}$ values in the learning set S of elements located at this node are calculated, and the value of $a_{c}$ is drawn from the set $\left[\left.a_{min}^{S},a_{max}^{S}\right]\right.$ according to the uniform distribution. The partition rule $s_{i}$ created in this way is then $a_{i}<a_{c}$. If, alternatively, $a_{i}$ is a categorical variable with values in the set A, then the set $A_{S}$ of unique values of this variable occurring in the current learning set S is determined. Then, a non-empty subset $A_{1}\subset A_{S}$ and a subset $A_{2}\subseteq A\setminus A_{S}$ are drawn. The $s_{i}$ partition rule thus determined is $a_{i}\in A_{1}\cup A_{2}$. After calculating K rules for all drawn variables, the selected rule $s^{*}$ is the one with the highest Score coefficient. For a given partition rule s and set S, it is calculated as

(13) $$Score_{c}\left(s,\;S\right)=\frac{2I_{c}^{s}\left(S\right)}{H_{s}\left(S\right)+H_{c}\left(S\right)},$$

where $I_{c}^{s}\left(S\right)$ is the mutual information resulting from the classification and the created rule, $H_{s}\left(S\right)$ is the entropy of the partition rule, and $H_{c}\left(S\right)$ is the entropy of the classification. The selected rule $s^{*}$ divides the set S into $S_{l}$ and $S_{r}$—sets of elements belonging to the left and right nodes created by this rule. Subsequent rules are created using the aforementioned sets and are attached to the corresponding nodes in the decision tree [59].

The extra trees algorithm has been used in the past for the purposes of classifying gas–liquid two-phase flow patterns [60], predicting the equilibrium CO_2_ loading capacity in aqueous solutions of adsorbents [61], and predicting the thermal performance of buildings with roofs made of phase-change materials [62].

## 3. Materials and Methods

The sequencing batch reactor with activated sludge is an alternative to continuous flow activated sludge bioreactors. Three identical reactors (SBRs) with a total volume and effective volume of 10 dm^3^ and 8 dm^3^, respectively, were used in this study. The SBRs were inoculated with activated sludge and supplied each cycle with the raw wastewater from the secondary settling tank, both media coming from the Hajdów Municipal Wastewater Treatment Plant (WWTP) in Lublin (south-eastern Poland). The WWTP daily flowrate was ca. Q_d_ 60,000 m^3^·d^−1^. The operation time of each SBR was 12 h per cycle: 0.5 h for filling, 2 h for mixing, 7 h for aeration, 1.5 h for settling, 0.5 h for decanting, and 0.5 h for idle phase. The volumetric exchange ratio was maintained at ca. 35%. Air supply was dispersed at the bottom, and the aeration rate was adjusted by a rotameter. Operating temperature was maintained at 20 °C ± 0.1 °C, and dissolved oxygen (DO) at 2 gO_2_/m^3^ in each reactor. In the experiment, the parameters of the activated sludge used were as follows: SRT = 15 d (sludge retention time), F/M ratio = 0.10 gBOD_5_/gMLVSS·d (food-to-microorganism ratio), MLSS = 3.2 g/dm^3^ (mixed liquor suspended solids), and SVI = 235 mL/g (sludge volume index).

Following the addition of raw wastewater to the bioreactor, there was a decrease in sensor resistance, which resulted from the highly polluted air sampled from the headspace, in comparison to the clean air utilized for flushing. The first operational phase of the SBR was 2 h of mixing. At the beginning of the mixing of raw wastewater, supernatant water, and activated sludge, the quantity of gaseous pollutants contained in the air markedly increased, which contributed to a sudden decrease of sensor resistance. The following phase consisted of the sequential aeration of the reactor, which lasted for 7 h, and was followed by 1.5 h sedimentation and 0.5 h decantation. Despite a relatively high variability of physicochemical parameters in the raw wastewater, continuous monitoring indicated multiple recurring cycles, bearing a close resemblance to optimal bioreactor operation. Irregularities in the typical characteristics correspond to the changes in bioreactor operation (possibly a failure of an SBR or part thereof) or reduced efficiency of wastewater treatment, which may stem, for example, from the influx of substances that are harmful to the activated sludge.

**Figure 1:**
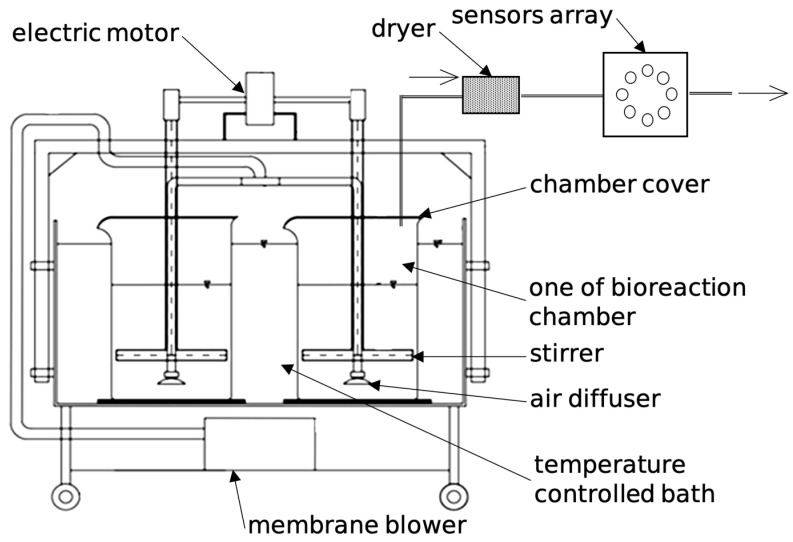
Schema of SBR and measurement system.

The measurement of the gas sample using the matrix of sensors covered all stages of the normal operation of the SBR bioreactor, shown in Figure 1, and included the following phases: filling, mixing and aeration, sedimentation, and decantation.

The flowrate of the sample stream was constant and amounted to 200 cm^3^/min. The array was flushed with clean air during the decanting of the SBR tanks. The measurement lasted 60 days, during which 120 SBR cycles were performed, and the measurement data were recorded with a frequency of 1 Hz. Gas samples were dried with a Perma Pure LLC DM-110-24 membrane Nafion tube dryer with silica gel (New Hampshire Ave, NJ, USA). The measurements resulted in 611 observations of multivariate data collected during the experiment.

During the 60 days, signal drift was noticeable due to the slow contamination of sensors operating under harsh conditions. The average change in absolute resistance for all sensors was 0.148 kΩ/day, with the lowest value observed for the S6 sensor (TGS2611 with carbon filter) and the highest for the S2 sensor (TGS2602).

The gas array used for the tests consisted of 8 MOS gas sensors, as detailed in Table 1, each with a different sensitivity and selectivity to tested chemical compounds [63]. Additionally, thermal and humidity parameters of the sample were monitored. For temperature measurements, the digital DS18B20 Maxim Integrated -Dallas sensor (San Jose, CA, USA) was applied (range, −55 °C to +125 °C; accuracy, ±0.5 °C from −10 °C to +85 °C) [64]. In the case of humidity, the HIH-4000 Honeywell (Minneapolis, MN, USA) relative humidity sensor was used (range, 0–100%; accuracy, ±3.5% RH) [65].

**Figure 2:**
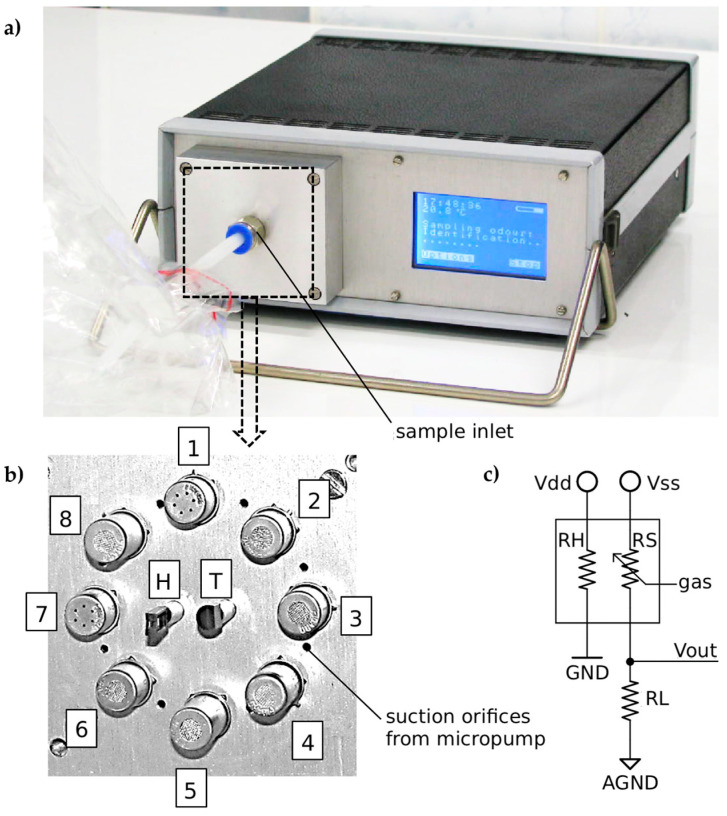
E-nose with 8 MOS sensors: (**a**) view of device during measurement; (**b**) view of sensor upon front cover, where (1) TGS2600-B00, (2) TGS2610-C00, (3) TGS2611-C00, (4) TGS2612-D00, (5) TGS2611-E00, (6) TGS2620-C00, (7) TGS2602-B00, and (8) TGS2610-D00, T—DS18B20, H—HIH-4000; (**c**) schema of sensor connection.

The sensors enclosed in the sensor chamber are arranged in a circular array (Figure 2). The measured gas is sampled through the port located in the central part of the sensor chamber housing, and the orifices located between all the sensors in the rear wall of the chamber enable even distribution of the gas sample.

A diagram of the measuring system of the MOS sensor is shown in Figure 2c. The circuits of the RH heaters and RS sensor elements are powered by 5V DC from separate voltage stabilizers. The sensory elements of the sensors change their resistance depending on the concentration of the gas they are designed to detect. In order to determine the resistance of the MOS sensor, a voltage divider was used, and the value of the RL resistor was selected depending on the range of variation of the sensor element’s resistance. The resistance of the sensor is calculated with the following equation: $R_{S}=R_{L}\cdot \left(V_{SS}-V_{OUT}\right)\cdot {\left(V_{OUT}\right)}^{-1}$, where $R_{S}$ is the resistance of the sensor [Ω], $R_{L}$ is the resistance of the load resistor [Ω], $V_{SS}$ is the voltage reference of the resistor divider [V], $V_{OUT}$ is the output voltage of the resistor divider [V]. The output voltage is measured using a 24-bit Analog Devices ADuC847 (Wilmington, MA, USA) analog-to-digital converter.

Sampling is possible with the built-in membrane micropump. In addition, the measuring device is equipped with a graphic display with a touch panel and a battery. Measurement data are saved on an SD memory card.

The data analysis was performed in Jupyter Notebook [66], with the Python programming language [67]. During the research, fundamental packages, such as pandas [68] or numpy [69], were used. Seaborn [70], matplotlib [71], and plotly [72] were responsible for the visualization part, while sklearn [50] provided PCA, DBSCAN, and extra trees classifier methods, among others.

## 4. Results

At the beginning of the analysis, the records with missing values were removed. The dataset contains 611 observations and 9 variables, 1 categorical (stage) and 8 numerical (measurements from sensors). For further analysis, the numerical variables were standardized.

**Figure 3:**
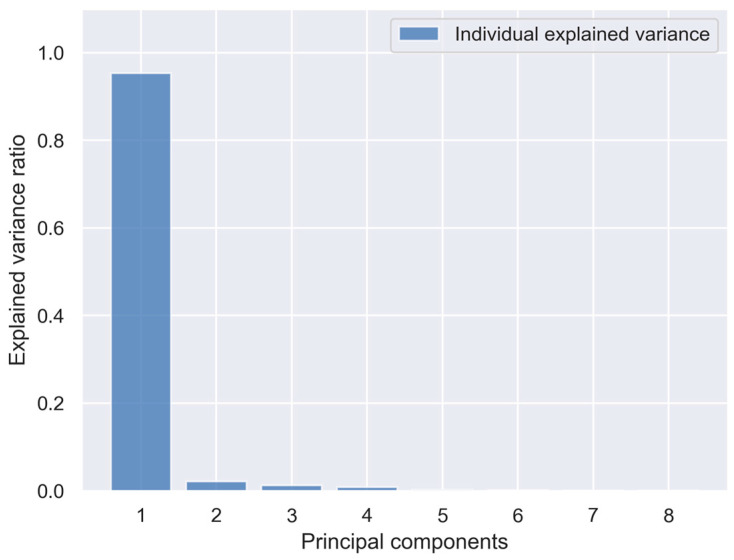
Individual explained variance by each of the principal components.

In order to visualize the multidimensional data, a principal component analysis was performed. According to the criterion of explained variance, such a number of principal components should be selected to exceed a certain threshold of the cumulative explained variance. In Figure 3, the bars represent the percentage of explained variance by each of the principal components. It can be seen that the first principal component (PC1) already provides over 95% of the explained variance. That is why even the first component exceeds the threshold of 90% of cumulative explained variance, which is the highest of the widely adopted cut-offs.

**Figure 4:**
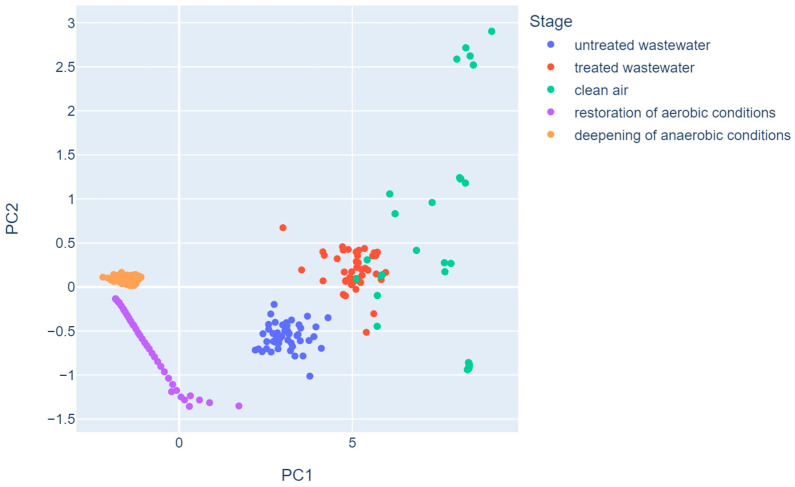
Two-dimensional PCA mapping of the data.

However, in order to visualize the data in a two-dimensional plane, first two principal components have been chosen. Figure 4 depicts data in a two-dimensional PCA plane, and observations are colored using the unique values of the stage variable. It can be found that only the observations from clean air samples overlap point clouds of other stage variable values on the graph. The most concentrated group of observations are those from the deepening of anaerobic conditions, while treated wastewater forms a point chain on the graph.

An experimental malfunction of the SBR was simulated and carried out at the beginning of the study. It consisted in turning off the mixing and aeration systems, which created conditions that were conducive to the development of anaerobic bacteria. The resistances of all sensors were markedly changed. The resistance of the sensors calculated from the daily median for the failure recovery phase (day 3) in relation to the median resistance in the following days (≥4) of normal operation is on average 26%, respectively, for individual sensors in the following order: 21%, 25%, 27%, 29%, 23%, 38%, 28%, and 21%. This step was called “deepening of anaerobic conditions”. Subsequently, the aeration and mixing systems were turned back on. There was an increased release of noxious gases (with high olfactory nuisance) as a result of the activated sludge operation under anaerobic conditions. Since the air in the wastewater headspace was significantly contaminated, the resistance of sensors was reduced in comparison to the normal conditions. The conditions that characterize the standard mode of operation were progressively restored in consecutive runs. Figure 4 shows that two deviations from normal bioreactor operation can be identified with ease. This proves that gas sensor arrays can be successfully used to continuously monitor the bioreactor conditions, providing instant notifications about the irregularities in operation. Since the sensors are not immersed in wastewater (i.e., a harmful environment), they exhibit higher durability compared to the sensors employed in immersive methods.

**Figure 5:**
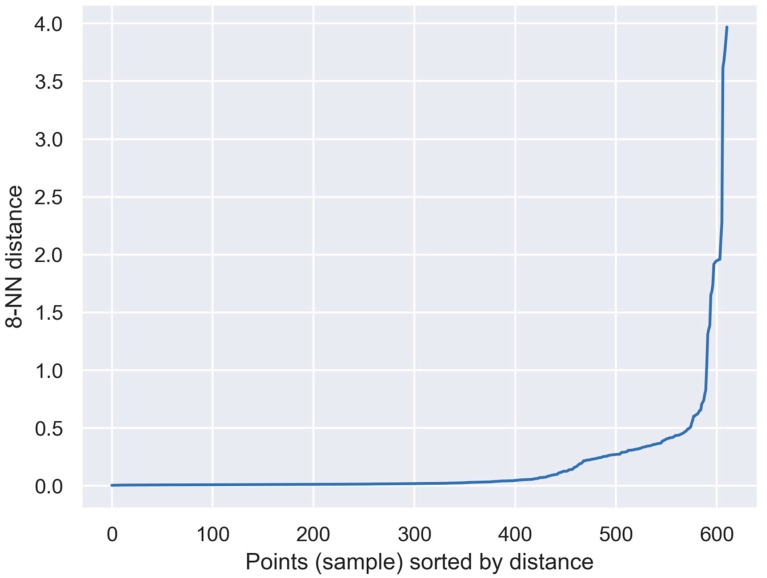
Distance plot of k-NN method with *k* = 8.

A cluster analysis was performed, using the DBSCAN algorithm. For the purpose of choosing the best parameters for the analyzed task, the k-nearest neighbors method was applied. In this method, it is recommended to use the number of dimensions of the analyzed data as the k parameter. The eps parameter for DBSCAN, which is the ε mentioned in Equation (4), is estimated at the bend of the k-NN distance plot presented in Figure 5, and the algorithm was performed for its value equaling 0.5.

**Figure 6:**
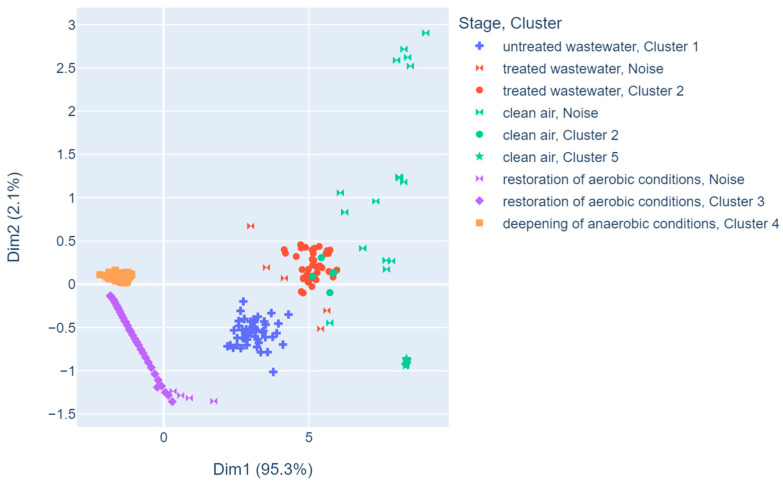
DBSCAN clustering results with the dimensions on axes created with PCA method.

DBSCAN grouped the data into five different clusters, distinguishing them from the noise points. The result of the algorithm is shown in Figure 6. It is worth mentioning that while the clusters are not identical to the original sample classes, they are still very similar. Two groups that are clearly separated from each other are Cluster 1 and Cluster 2. DBSCAN correctly separated the class of untreated wastewater from treated wastewater. It placed all the observations from untreated wastewater in Cluster 1, while those from treated wastewater were placed in Cluster 2. Some of the data from the treated wastewater samples were classified as noise, but there was no addition of data from either class to the cluster containing the data of the other. Similarly, Cluster 3 and Cluster 4 are separated, too. Again, the algorithm distinguished two original sample categories—restoration of aerobic conditions and deepening of anaerobic conditions. Only some observations from restoration of the aerobic conditions are denoted as noise. Cluster 3 is the most noteworthy, since the DBSCAN algorithm, which calculates the distances between points, attaches them in a chain fashion to the current cluster. With another non-hierarchical clustering algorithm, these observations might not be assigned to a single cluster, since the points are not concentrated in a spherical way. The clean air class was generally recognized as noise points, and only a few of these observations were assigned to Cluster 2 and Cluster 5.

In order to assess the DBSCAN grouping, the clustering quality measures were calculated. The values of the measures in Table 2 suggest a very good DBSCAN clustering quality.

The homogeneity score of 0.935 indicates a homogeneous grouping, while the completeness value (0.897) implies that almost all points belonging to a specific class are members of the same cluster. The V-measure result (0.916) confirms these conclusions. The outcomes of adjusted Rand index (0.988) and adjusted mutual information (0.914) also suggest a high quality of the DBSCAN clustering. Furthermore, the level of the silhouette coefficient (0.690), given that it always reaches values between −1 and 1, shows that the clusters are sufficiently well defined.

The last stage of the research was fitting an extra trees model to classify the stage variable. The data set was divided into the training and test set randomly, with the test set size equal to 25% of the number of all observations. The optimal parameters for the model were obtained by cross-validated grid search with 10 folds. The search regarded the following parameters:

- n_estimators—number of trees trained in algorithm;
- min_samples_leaf—minimum number of observations to form a leaf node in a tree;
- max_features—number of variables drawn at each node, which are then used for creating a split.

Table 3 presents the details of this search and obtained optimal values of parameters.

**Figure 7:**
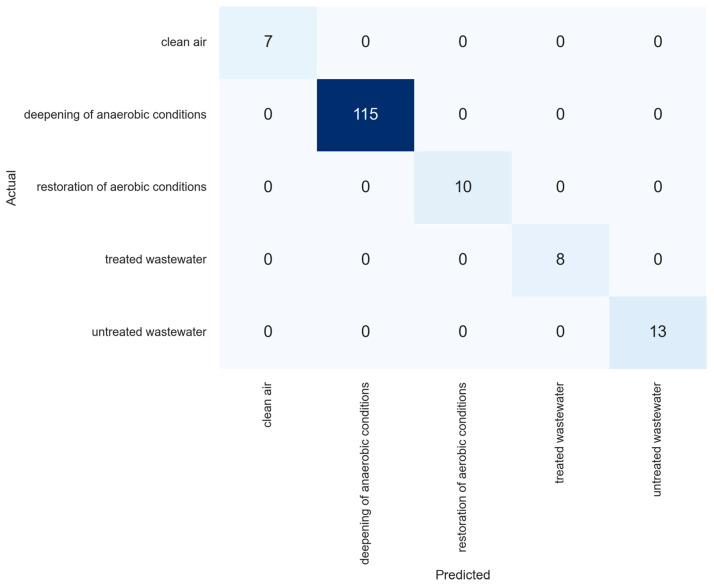
Contingency matrix for extra trees model on the test set. Greater blue saturation indicates a large number of observations in groups described in the matrix.

The extra trees model with the above-mentioned optimal parameters achieved 100% accuracy on the test set, correctly classifying each observation. Figure 7 presents the contingency matrix for the discussed classification problem.

## 5. Discussion

It is difficult (and not entirely expedient) to discuss and compare the results obtained from the operation of the SBR in the aspect addressed in this paper with literature reports, emphasizing the fact that the wastewater treatment process can be viewed as a dynamic system with balanced inflows and outflows. In addition, the authors are not aware of any other work in which the aforementioned methods were applied to analyze data describing, for example, a controlled failure of the aeration system. Thus, the discussion of the results is based on available articles (including the authors’) using the same methods of analysis, on possibly similar data sets.

In the work [36], the analyzed data came from an electronic nose equipped with eight MOS sensors, where the samples were divided in relation to the five classes of the SBR operation phases. These readings were taken in the bioreactor headspace using an electronic nose, the sensors of which responded to changes in air quality. Such an action allows early identification of failures and detection of anomalies in the wastewater treatment process. The authors obtained 98.2% cumulative explained variance when two principal components were selected. In the PCA mapping figure, the treated and untreated wastewater classes overlap, while the other groups form homogeneous groups. In article [45], this method was used to visualize measurements from the e-nose of buildings with varying degrees of mold bloom and reference samples. The two principal components used to create the graph collectively achieved nearly 85% of the explained variance. However, groups of point clusters formed on the plane did not create homogeneous clusters with respect to their mold infestation; only in the case of reference samples could such clusters be noticed. The authors of the paper [46] applied the PCA method to two sets containing data on the values of concentrations of chemical indicators at different locations. For both sets, the first two components did not reach a high percentage of the cumulative explained variance. As for the set containing 1104 observations, it was 39.9%, while for the set with 92 observations, it reached almost 56%. The PCA score plots for both instances were illegible and did not allow separating clusters of objects. Bourgeois et al. in paper [73] presented an analysis using the PCA method, in which the classes resulting from disturbances in the wastewater quality caused by pollution episodes or other abnormal events during treatment were clearly separable in the plot. In the article [17], gas fingerprint data helped distinguish different water samples from reference samples and identify those in which quality deviated from the reference based on PCA analysis.

The results obtained in this work resemble those obtained in [36,45,73], as the PCA method was used to present the dataset in a two-dimensional graph and the percentage of explained variance by the two components was high (over 95%), which enables one to clearly distinguish between groups of observations from different operating stages of the bioreactor with activated sludge. In addition, most of the data formed homogeneous clusters, while the other overlapped with groups formed by different stage classes.

The DBSCAN non-hierarchical clustering algorithm in the paper [56] was applied to group the areas belonging to a municipal water supply network into homogeneous zones. The analyzed data concerned leakage characteristics of the pipe system. The group labels thus determined were used to create a model for detecting leakage in the system. Another application of this algorithm is the detection of outlier data, referred to as noise in this method. Such an approach was covered in the article [57], in which the data from IoT sensors, before applying the random forest model, was refined from the data unclassified into any group in DBSCAN. On the other hand, the groups created with DBSCAN in [58] were used to compare them to the true labelling of the data in the groups of anomaly occurrence and normal operation of wastewater treatment plants.

In the present study, DBSCAN was applied in similar manner to that presented in [58], as whether the clusters coincide with real data categories was considered. Moreover, DBSCAN managed to correctly cluster both data distributed around a certain core point and data arranged in a chain. Since DBSCAN also allows certain points to be classified as noise, it can be assumed that some anomaly has occurred if they are present. The reasons for this should be sought and confirmed by known reference methods. Two groups that are clearly separated from each other are Cluster 1 and Cluster 2. DBSCAN correctly separated the class of untreated wastewater from treated wastewater. It placed all the observations from untreated wastewater in Cluster 1, while those from treated wastewater were placed in Cluster 2. Some of the data from treated wastewater samples were classified as noise, but there was no addition of data from either class to the cluster containing the data of the other.

The extra trees algorithm from paper [61] was trained for a regression task of predicting equilibrium absorption of CO_2_ in solvents and achieved an R^2^ equal to 0.9995 on training and 0.9982 on the test set. A different regression task for which this algorithm was applied is presented in [62]. The authors predicted the thermal performance of materials, and the coefficient of determination on the test data reached 0.9456. On the other hand, in the work [60], the extra trees classifier was applied for recognition of gas–liquid flow regime classes in S-shaped pipeline risers. This model had a classification accuracy of 82.41%.

The result obtained in the above-mentioned article is similar to the outcome of the present work, as the task for the model is classification and accuracy of the model is high. However, the accuracy of the classification in the current work is better, as the model achieved perfect performance on the test set. Such a good classification is probably due to the fact that the parameters of the wastewater in the bioreactors differed, which were: untreated, treated wastewater, bioreactors in the failure phase, and with the correct technological parameters of operation restored.

## 6. Summary and Conclusions

In standard small-scale wastewater treatment plants, classical measurements are seldom conducted, and the staff often relies on their own experience and their own senses to control processes. The proposed system for assessing the performance of bioreactors based on a gas sensor array, as well as an appropriately selected procedure for visualization and analysis of multidimensional data, can be a good complement and help in the operation of small-scale treatment plants that do not have specialized physico-chemical laboratories. Simultaneously, similarly to the experience of skilled staff, the models that analyze measurement data can be developed on the basis of subsequent observations. Moreover, unlike the human olfactory sense, the e-nose does not adapt to noxious odors and can be quickly regenerated by briefly flushing the gas sensor arrays with clean air.

Analysis of visualizations and results from data mining models allows the following conclusions:

- Principal component analysis allows one to distinguish observations related to deviations and normal bioreactor operation, while the first two principal components explained over 95% of variance. However, not all stages are desegregated, as some of them overlap in the plot.
- The density-based clustering method DBSCAN managed to cluster the data into five groups, which is the same number as the true number of stage classes. However, not all observations were classified into the appropriate clusters.
- Although the restoration of the anaerobic conditions class arranged itself into a chain of points on the graph, owing to the ability of the DBSCAN algorithm to group data arranged into different shapes (not just spherical), the algorithm joined these observations into a single cluster. In addition, different clustering measures confirm that clustering with this algorithm was of good quality.
- Some observations from the classes of treated wastewater, clean air, and restoration of aerobic conditions were classified by DBSCAN as noise. Such an occurrence may herald the occurrence of an abnormal situation in the bioreactor and should be investigated for failure prevention.
- The extra trees supervised learning algorithm performed much better on the task of classifying objects into the appropriate classes. With optimal values of grid search parameters, it achieved 100% classification accuracy on the test set.

## Figures and Tables

**Table 1:** Overview of the gas sensors (Figaro USA Inc., Rolling Meadows, IL, USA) implemented in the e-nose [63].

Sensor ID	Type and Manufacturer	Description and Technical Parameters
1	TGS2600-B00 Figaro	Gas sensor: general air contaminants, methane, CO, isobutane, ethanol, hydrogen; detection range, 1–30 ppm (for hydrogen); resistance, 10–90 kΩ for clean air.
2	TGS2602-B00 Figaro	Gas sensor: general air contaminants, VOC, ammonia, hydrogen sulfide, ethanol, toluene, odorous compounds; detection range, 1–30 ppm (for ethanol); resistance, 10–100 kΩ for clean air.
3	TGS2610-C00 Figaro	Gas sensor: LP gas and vapor detection, ethanol, hydrogen, methane, isobutane, propane. Butane; detection range, 500–10 k ppm; resistance, 0.68–6.8 kΩ for iso-butane.
4	TGS2610-D00 Figaro (with carbon filter)	Gas sensor: LP gas and vapor detection, ethanol, hydrogen, methane, isobutane, propane. Butane; detection range, 500–10 k ppm; resistance, 0.68–6.8 kΩ for iso-butane.
5	TGS2611-C00 Figaro	Gas sensor: methane, hydrogen, iso-butane, ethanol; detection range, 500–10 k ppm; resistance, 0.68–6.8 kΩ for methane.
6	TGS2611-E00 Figaro (with carbon filter)	Gas sensor: methane, hydrogen, iso-butane (uses filter material in its housing, which eliminates the influence of interference gases such as alcohol); detection range, 500–10 k ppm; 0.68–6.8 kΩ for methane.
7	TGS2612-D00 Figaro	Gas sensor: mostly LNG and LPG methane, propane, iso-butane, solvent vapors; detection range, 1–25% LEL; resistance, 0.68–6.8 kΩ for methane.
8	TGS2620-C00 Figaro	Gas sensor: alcohol, solvent vapors; detection range, 50–5 k ppm; resistance, 1–5 kΩ for ethanol 300 ppm.

**Table 2:** Summary of clustering quality measures of DBSCAN algorithm.

Clustering Quality Measure	Value
Homogeneity	0.935
Completeness	0.897
V-measure	0.916
Adjusted Mutual Information	0.914
Adjusted Rand Index	0.988
Silhouette Coefficient	0.690

**Table 3:** Grid search details for extra trees model.

Parameter	Vector of Checked Values	Optimal Value
n_estimators	[50, 100, 200]	50
min_samples_leaf	[2, 5, 20]	2
max_features	[2, 5, 8]	8

## Data Availability

All important data are available in the paper.
